# Machine Learning: An Approach in Identifying Risk Factors for Coercion Compared to Binary Logistic Regression

**DOI:** 10.3389/fpsyt.2018.00258

**Published:** 2018-06-12

**Authors:** Florian Hotzy, Anastasia Theodoridou, Paul Hoff, Andres R. Schneeberger, Erich Seifritz, Sebastian Olbrich, Matthias Jäger

**Affiliations:** ^1^Department for Psychiatry, Psychotherapy and Psychosomatics, University Hospital of Psychiatry Zurich, Zurich, Switzerland; ^2^Psychiatrische Dienste Graubuenden, Chur, Switzerland; ^3^Universitaere Psychiatrische Kliniken Basel, Universitaet Basel, Basel, Switzerland; ^4^Department of Psychiatry and Behavioral Sciences, Albert Einstein College of Medicine, New York, NY, United States

**Keywords:** coercion, seclusion, restraint, coercive medication, involuntary hospitalization, machine learning

## Abstract

**Introduction:** Although knowledge about negative effects of coercive measures in psychiatry exists, its prevalence is still high in clinical routine. This study aimed at define risk factors and test machine learning algorithms for their accuracy in the prediction of the risk to being subjected to coercive measures.

**Methods:** In a sample of involuntarily hospitalized patients (*n* = 393) at the University Hospital of Psychiatry Zurich, we analyzed risk factors for the experience of coercion (*n* = 170 patients) using chi-square tests and Mann Whitney U tests. We trained machine learning algorithms [logistic regression, Supported Vector Machine (SVM), and decision trees] with these risk factors and tested obtained models for their accuracy via five-fold cross validation. To verify the results we compared them to binary logistic regression.

**Results:** In a model with 8 risk-factors which were available at admission, the SVM algorithm identified 102 out of 170 patients, which had experienced coercion and 174 out of 223 patients without coercion (69% accuracy with 60% sensitivity and 78% specificity, AUC 0.74). In a model with 18 risk-factors, available after discharge, the logistic regression algorithm identified 121 out of 170 with and 176 out of 223 without coercion (75% accuracy, 71% sensitivity, and 79% specificity, AUC 0.82).

**Discussion:** Incorporating both clinical and demographic variables can help to estimate the risk of experiencing coercion for psychiatric patients. This study could show that trained machine learning algorithms are comparable to binary logistic regression and can reach a good or even excellent area under the curve (AUC) in the prediction of the outcome coercion/no coercion when cross validation is used. Due to the better generalizability machine learning is a promising approach for further studies, especially when more variables are analyzed. More detailed knowledge about individual risk factors may help to prevent the occurrence of situations involving coercion.

## Introduction

The use of coercive measures (e.g., seclusion, physical and mechanical restraint, forced medication) in psychiatric patients is a massive invasion in their integrity and freedom. As a result, the usage of coercion is controversially discussed since the beginning of modern psychiatry and certain approaches have tried to reduce its rates (1). Although some of those approaches were successful, there are still many patients in which coercion is used. Often the usage of coercion seems necessary when the patients are a danger for themselves or for others due to an underlying psychiatric disorder (2, 3). These situations are always associated with an ethical dilemma. On one side coercion shall help to protect the patient's or other's integrity (2, 3). On the other hand it restricts the freedom of the person which is one of the basic human rights (4). Being a threat to oneself or others may have different reasons in psychiatric patients. In some situations patients are delusional and feel threatened by others which leads to the reaction to protect themselves and can result in threats to other patients or staff (5). Also in situations where the patients are threatening themselves or have suicidal ideations caused by the symptoms of their psychiatric disorder, coercive measures might become necessary to secure the patients survival.

The use of coercion distinguishes psychiatry from other medical disciplines where informed patients can decide to accept or reject a specific measure. Psychiatry at one hand aims to help the patients to develop a self-determined life without burden of psychiatric symptoms. On the other hand psychiatry is legally determined to reject the patients freedom to move (involuntary hospitalization) but also the freedom to reject a specific measure (forced medication, physical or mechanical restraint, seclusion) if harm to self or others has to be disrupted.

It is obvious that such situations are challenging for the patients but also for the therapeutic team. Those challenges were topic of previous studies where it was shown that patients who experienced coercive measures often describe feelings of helplessness (6, 7), fear (8), anger (9, 10) and humiliation (11). Due to that, some patients stated to avoid searching for psychiatric help in a crisis (12, 13). On the other hand there were some patients who retrospectively agree with the coercive measure (7, 9) and state that they would like to be forced into treatment again in the case of a future crisis (14). These contrary findings underline the controversy of this topic.

It was the goal of earlier studies to understand which patients experience coercion and to characterize their clinical, but also their socioeconomic features. Gaining better understanding of risk factors to experience coercion was thought to be helpful in the development of therapeutic strategies for patients at risk and thus, to reduce the prevalence of coercion.

During the last years specialized psychiatric intensive care units (PICU) had been the center of extensive research and it could be shown that some patient characteristics are associated with the transfer from a general psychiatric unit to a PICU and with the usage of coercion on these specialized wards (15). Furthermore psychotic disorders were shown to be frequently associated with coercion (16–24). Also personality disorders (25, 26), substance-use-related disorders (19) and mental retardation (25) were found to be associated with coercion. A history of aggression (16–18, 22, 23, 25, 27–29) was frequently found to be associated with coercion and violence/threats were described to be the second most frequent reasons after agitation/disorientation for the usage of coercion (30). Patients with a history of former voluntary and/or involuntary commitments (IC) and frequent hospitalizations (16–20, 24) and those with longer duration of hospitalizations (31) were also described to experience coercion more often. Those factors were described nearly uniformly throughout literature. Whereas other factors like male (20, 23–25, 32, 33) and female gender (22, 29) or younger (19, 20, 23, 25, 28, 29, 32, 33) and older age (22, 24) were controversially associated with coercion in different study sites. These inconsistent findings impede the definition of risk-factors which are independent of specific countries. The inconsistencies between study sites were discussed to be caused by cultural influences, organizational factors, societal factors, the clinic-culture or a combination (34, 35). Besides that, one has to bear in mind that prior studies followed different methodological approaches to analyze data which additionally limits the comparability between different study sites. Some studies used descriptive approaches (16, 32) or group comparisons with binominal, non-parametric tests or ANOVA (17–20, 22–24, 26, 29, 30). To describe risk factors regression analysis was frequently used (19–21, 23, 26, 28, 29, 31, 33) and some studies extended their findings with an estimation of the area under the curve (AUC) (23). One study used a latent class analysis (LCA) which is capable of detecting the presence of groups in individuals with relatively homogeneous clinical courses (25). Another study used Multilevel random effects modeling (27). Only a few studies tried to describe the potency of specific risk factors to affect the outcome coercion/no coercion. Furthermore, the description of the specificity and sensitivity of the statistical models is scarce. One study which followed this approach described an acceptable AUC for one model using bivariate analysis (23). Another study found that with the included parameters only a limited prediction of patients at risk was possible (31). Thus, besides the analysis of risk factors at our study site, the second aim of this study was to find statistical approaches with a good balance in their specificity and sensitivity and prediction accuracy for the outcome “coercion/no coercion” in psychiatric inpatients. Furthermore we wanted to analyze the risk factors for their weights in affecting the outcome coercion/no coercion.

In today's psychiatric research machine learning is an emerging methodology. It is connoted with a great potential for innovation and paradigm shift as the algorithms facilitate integration of multiple measurements as well as allow objective predictions of previously “unseen” observations. We used this new approach to train and compare models with parameters available at admission and after discharge. To test for the hypothesis that machine learning algorithms are effective in the prediction of the outcome coercion/no coercion in psychiatric patients we compared binary regression analysis to the machine learning algorithms according to their sensitivity, specificity, accuracy, and AUC. Furthermore, we used machine learning to weight the included predictors for their potency in affecting the outcome coercion/no coercion. For the comparison of the two approaches we analyzed clinical data of involuntarily hospitalized patients at the University Hospital of Psychiatry Zurich and built two groups depending on the outcome Coercion/No Coercion.

## Methods

### Setting

The study was reviewed and approved by the Cantonal Ethics Commission of Zurich, Switzerland (Ref.-No. EK: 2016-00749, decision on 01.09.2016). Commitment documents as well as the medical records of patients involuntarily hospitalized at the University Hospital of Psychiatry Zurich during a 6-month period from January first to June 30, 2016 were analyzed.

*N* = 16 wards of the University Hospital of Psychiatry Zurich with a total of 252 beds were included. The clinic provides mental health services for a catchment area of 485,000 inhabitants.

### Study sample

No exclusion criteria were defined. We screened a comprehensive cohort of all patients admitted voluntarily and involuntarily to the University Hospital of Psychiatry Zurich during a 6-month period from January first to June 30, 2016 (*n* = 1,699 patients). For the analysis we included involuntarily committed patients (*n* = 577) and voluntarily committed patients who were retained at a later stage during their hospitalization and then changed to the legal status of involuntary hospitalization (*n* = 35).

### Selection of predictor variables

Selection of predictor variables for “training” an algorithm in machine learning is challenging. We used a recommended method and searched the literature databases for variables which were already described to be associated with the usage of coercion: Psychiatric diagnosis (16–24), aggressive behavior (16–18, 22, 23, 25, 27–30), former voluntary or involuntary commitment (IC) and frequent hospitalizations (16–20, 24), gender (20, 22–25, 29, 32, 33), and age (19, 20, 22, 23, 25, 28, 29, 32, 33) were identified as variables of interest. We searched the routine documentation in the electronic medical files of the patients for these variables. The medical files include documentation about the socio-demographic parameters, admission circumstances, prescribed medication, documentation of coercive measures, and treatment planning. As there was no standardized assessment for aggression we searched which indirect information could be used and found IC due to danger to others and involvement of police in the admission process as indirect markers for aggressive behavior. Furthermore we included the procedural aspects abscondence, appeal to the court, duration until day passes, duration of IC, duration of hospitalization into analysis. When patients are exposed to coercive medication mostly antipsychotics or benzodiazepines are used. We were interested if the patients, exposed to coercion differed from those without coercion according to their regular prescribed medication during hospitalization. Thus, we searched the medical files for the prescription of medication classes (antipsychotics, antidepressants, benzodiazepines, and others).

### Analysis and machine learning

We conducted analysis with MATLAB (MATLAB and Statistics Toolbox Release 2012b, The MathWorks, Inc., Natick, Massachusetts, United States.) and SPSS 23.0 (IBM Corp. Released 2011. IBM SPSS Statistics for Windows, Version 23.0. Armonk, NY: IBM Corp.) for Windows.

In a first step we compared patients with/without experience of coercion. We used cross-tabulation with chi-square tests for categorical variables. Due to the non-normal distribution we used Mann–Whitney tests for numeric variables. Variables that differed between both groups in bivariate analyses were included as potential risk factors in multivariate analysis. To analyze the impact of the risk factors on the outcome coercion/no coercion binary logistic regression analysis was used with coercion/no coercion as the dependent variable. The goodness of fit of the binary logistic regression model was assessed by the receiver operating characteristic (ROC) curve method. The AUC served as the criterion to determine the level of discrimination. Discrimination was deemed acceptable at AUC values between 0.7 and 0.79, excellent at values between 0.8 and 0.89, and outstanding at values over 0.9 (23). The specificity and sensitivity, positive predictive value (PPV) and negative predictive value (NPV) were calculated from the results of the different models.

Because of multiple comparisons Bonferroni's adjustments were made to prevent Type I error inflation (α = 0.05/5 = 0.01).

In a second step we tested the hypothesis that machine learning algorithms can be used to predict the outcome. Again the outcome of coercion/no coercion was used as dependent variable. Because the outcome was already defined, supervised learning algorithms [Logistic regression, supported vector machine (SVM), and bagged trees algorithms] were used. We used cross-validation to test the trained model. The training set was divided in 5 equal sized subsets with one part being used to train a model and the other four subsets to evaluate the accuracy of the learnt model (five-fold cross validation). The error rate of each subset was an estimate of the error rate of the classifier. Cross-validation is used in machine learning to establish the generalizability of an algorithm to new or previously “unseen” subjects. The validity of the algorithms in predicting the outcome coercion from no coercion was evaluated using prediction accuracy, sensitivity, specificity, positive predictive value (PPV) and negative predictive value (NPV). In this study, sensitivity and specificity represented correctly predicted occurrence of coercion (true positives) and correctly predicted lack of coercion (true negatives), respectively.

### Logistic regression

The classifier models the class probabilities as a function of the linear combination of predictors. Logistic regression utilizes a typical linear regression formulation.

### Support vector machines (SVM)

This technique separates data by a hyperplane, trying to maximize the margin and creating the maximum distance between the hyperplane and the values which lie on each side. The higher this distance gets the better is the reduction of the expected generalization error.

SVM are robust in dealing with large numbers of features included because only those features which lie on the margin of the hyperplane are included. If data are non-linear and separation is not possible on one hyperplane, SVM can create more dimensional hyperplanes in a higher dimensional feature space. SVM methods are binary. So in the case of this study where we compared the patient group with/without coercion no dummy-variables had to be created for the response-feature.

### Decision trees

Decision trees classify instances by sorting them based on feature values. The nodes represent instances in the feature to be classified and the branches represent values that the node can become. The instance which divides the training data in the best way is selected as the root node. Than the instance which best divides this feature is chosen and so on. There are many ways to select the instance which is best at dividing data. It is possible to train ensembles of regression trees. They combine results from many weak learners into one high-quality ensemble model and are potent in the analysis of skewed data.

In generally, methods like SVMs and neural networks perform well with balanced continuous and multi-dimensional features whereas logic-based systems like decision trees or rule learners perform better with discrete/categorical variables.

SVMs are potent in dealing with large data which increase their prediction accuracy. These techniques can also work in the case of multi co-linearity and non-linear relationships. Logic based systems like decision trees are easier to interpret than SVMs.

### Imbalance problem

Class imbalance where the number of patients in one class (e.g., no coercion) exceeds the patients in the other class (e.g., coercion) is a common problem in machine learning. A typical machine learning algorithm trained with an imbalanced data set would assign new observations to the majority class (e.g., no coercion) (36). In this study we met this problem by creating an artificial group with balanced distribution of the outcome (coercion/no coercion). We assigned random numbers to the cohort of 612 patients which were involuntary hospitalized during the study period. We selected those patients without documentation of coercion during their hospitalization and sorted them by ascending numbers. We then excluded the first half of this group of patients. Thus, we conducted the analysis with 393 patients (no coercion: *n* = 223, coercion: *n* = 170). In those patients who experienced coercion, at least one coercive measure (e.g., seclusion, coercive medication, restraint alone, or in combination) was used during hospitalization.

## Results

### Comparison between groups of patients with/without coercion during involuntary hospitalization

Being a threat to others (72%) or self and others (20%) were the most frequent reasons for the usage of coercion. Clinical aspects like a higher CGI at admission, psychotic or personality disorders, the prescription of antipsychotics and benzodiazepines, harm to others or harm to self and others before admission, and male gender were significantly associated with the usage of coercion. From the procedural side being retained, police involvement at admission, the number of former admissions, a history of IC, a longer duration until patients were allowed for day passes, duration until revocation of involuntary hospitalization and duration of hospitalization, appeal for prolongation from the clinic but also appeal for early discharge from the patient were significantly associated with the use of coercion. We found an association between a secondary diagnosis of a substance-use-related disorder and coercion which was not significant (for details see Tables 1, 2).

**Table 1:** Comparison of socio-demographic and clinical aspects in patients with/without coercion.

	**Total (*****n*** = **393)**		**No Coercion**		**Coercion**		**χ^2^**	d.f.^*^	***P*-value**
	**N**	**%**	**N**	**%**	**N**	**%**			
Gender							7.858	1	0.003
Male	204	52	102	46	102	60			
Female	189	48	121	54	68	40			
Reason for IC							50.253	3	<0.001
Harm to self	193	49	143	64	50	29			
Harm to others	87	22	29	13	58	34			
Harm to self and others	101	26	44	20	57	34			
Other	12	3	7	3	5	3			
ICD-10 primary diagnosis							59.746	6	<0.001
Organic disorder (F0)	71	18	44	20	27	16			
Substance use disorder (F1)	49	13	37	17	12	7			
Psychotic disorder (F2)	159	40	70	31	89	52			
Affective disorder (F3)	51	13	31	14	20	12			
Neurotic disorder (F4)	37	10	36	16	1	1			
Personality disorder (F6)	13	3	1	1	12	7			
Other	13	3	4	1	9	5			
ICD-10 secondary F1 diagnosis							4.695	1	0.021
No	307	78	183	82	124	73			
Yes	86	22	40	18	46	27			
CGI at admission							28.857	3	<0.001
1–2	5	1	5	2	0	0			
3–4	18	5	15	7	3	2			
5–6	161	41	108	48	53	31			
7–8	209	53	95	43	114	67			
Police involved at admission							11.978	1	<0.001
No	257	65	162	73	95	56			
Yes	136	35	61	27	75	44			
Antipsychotics							50.147	1	<0.001
No	78	20	72	32	6	3			
Yes	315	80	151	68	164	97			
Benzodiazepines							25.006	1	<0.001
No	92	23	73	33	19	11			
Yes	301	77	150	67	151	89			
Retainment							19.167	1	<0.001
No	362	92	217	97	145	85			
Yes	31	8	6	3	25	15			
Former IC							22.197	1	<0.001
No	206	52	140	63	66	39			
Yes	187	48	83	37	104	61			
Abscondence									
No	317	81	195	87	122	72	15.203	1	<0.001
Yes	76	19	28	13	48	28			
Appeal for prolongation of IC							17.063	1	<0.001
No	354	90	213	95	141	83			
Yes	39	10	10	5	29	17			
Appeal for early discharge							14.257	1	<0.001
No	320	81	196	88	124	73			
Yes	73	19	27	12	46	27			
Rehospitalization during 6 months							12.951	1	<0.001
No	267	68	168	75	99	58			
Yes	126	32	55	25	71	42			

*CGI, Clinical Global Impression; IC, Involuntary Commitment. Chi-square test reveals significant differences between an involuntarily hospitalized cohort of patients which experienced coercion and those which did not experience coercion*.

**Table 2:** Comparison of socio-demographic and clinical aspects in patients with/without coercion.

	**Coercion**								**Mann–Whitney U**	***Z***	**Sig**
	**No**				**Yes**						
	**Min**	**Mean**	**Median**	**Max**	**Min**	**Mean**	**Median**	**Max**			
Number of former admissions	0	4	0	69	0	9	2	67	12468.500	−4.831	<0.001
Duration until revocation of IC	0	79	16	10	1	31	25	230	10937.500	−7.189	<0.001
Duration of hospitalization	0	138	22	13	1	37	31	245	11383.000	−6.789	<0.001
Duration until day passes	0	109	10	5	0	18	11	161	12468.500	−5.822	<0.001

*IC, Involuntary Commitment. Mann–Whitney U-Test reveals significant differences in procedural aspects of the cohort with compared to the cohort without coercion during hospitalization*.

Age at admission (Mann–Whitney U: 17454.000, Z: −1.346, *p* = 0.178, *n* = 393) and Nationality did not differ significantly between the groups [$\chi_{\left(6\right)}^{2}$ = 6.466, *p* = 0.373, *n* = 393]. Also we found no significant group difference for skills in German language, which is the official language in the state of Zurich, [χ^2^ = 0.384, *p* = 0.825, *n* = 393] and educational-level [$\chi_{\left(6\right)}^{2}$ = 8.285, *p* = 0.218, *n* = 393].

### Two models to predict the outcome coercion/no coercion

The main question of this study was to find models with a good accuracy in the prediction of the outcome coercion/no coercion. With a supervised learning technique a predictive model can be tested for both, input and output data. We trained and tested two models for their accuracy in the prediction of the outcome coercion/no coercion. For comparison we computed the same two models in binary logistic regression.

The first model included data which were available at hospital admission. In the second model we included variables which are available after a whole course of hospitalization. We hypothesized this second model to have higher prediction accuracy. The variables included in both models are shown in Table 3.

**Table 3:** Included predictors in both models.

**8 item model**	**18 item model**
1. Gender	1. Gender
2. Reason for IC	2. Reason for IC
3. Police involved at admission	3. Police involved at admission
4. ICD-10 primary diagnosis	4. ICD-10 primary diagnosis
5. ICD-10 secondary F1 diagnosis	5. ICD-10 secondary F1 diagnosis
6. Former admissions	6. Former admissions
7. Former IC	7. Former IC
8. CGI at admission	8. CGI at admission
	9. Retainment
	10. Antipsychotics
	11. Benzodieazepines
	12. Appeal for early discharge
	13. Appeal for prolongation of IC
	14. Abscondence
	15. Duration until day passes
	16. Duration until revocation of IC
	17. Duration of hospitalization
	18. Rehospitalization during 6 months

*IC, Involuntary Commitment, CGI, Clinical Global Impresssion*.

Binary logistic regression in SPSS and logistic regression in ML had the same results for B, SE, and p. This is comprehensible as logistic regression utilizes a typical linear regression formulation. The calculation of the coefficients/weights is different between both approaches and led to different results. Details are shown in Table 4.

**Table 4:** Findings of binary logistic regression and ML logistic regression.

**Factor**	B^*^	SE^*^	P^*^	tSTAT^**^	95% CI for Exp b^***^		
					**Lower**	**Exp B**	**Upper**
**8 ITEM MODEL**							
Gender	−0.432	0.235	0.066	−1.838	0.41	0.649	1.029
Former admissions	0.011	0.01	0.279	1.084	0.991	1.011	1.032
Former IC	0.591	0.256	0.021	2.310	1.094	1.806	2.982
Reason for IC	0.493	0.127	<0.001	3.878	1.276	1.636	2.099
Police involved at admission	0.466	0.244	0.056	1.910	0.988	1.594	2.571
CGI at admission	0.929	0.205	<0.001	4.521	1.692	2.532	3.787
ICD-10 primary diagnosis	0.098	0.067	0.141	1.473	0.968	1.104	1.258
ICD-10 secondary F1 diagnosis	0.206	0.279	0.46	0.739	0.711	1.229	2.124
**18 ITEM MODEL**							
Gender	−0.655	0.281	0.02	−2.330	0.3	0.52	0.901
Former admissions	0.012	0.012	0.321	0.991	0.989	1.012	1.036
Former IC	−0.122	0.312	0.696	−0.391	0.481	0.885	1.631
Retainment	2.142	0.56	<0.001	3.824	2.841	8.514	25.518
Reason for IC	0.556	0.157	<0.001	3.552	1.283	1.744	2.371
Police involved at admission	0.753	0.303	0.013	2.483	1.172	2.123	3.848
Rehospitalization during 6 months	0.127	0.301	0.672	0.424	0.63	1.136	2.048
Antipsychotics	1.569	0.5	0.002	3.138	1.802	4.802	12.795
Benzodieazepines	0.764	0.348	0.028	2.197	1.086	2.148	4.248
Duration until day passes	0.016	0.011	0.155	1.423	0.994	1.016	1.039
ICD-10 primary diagnosis	0.16	0.085	0.059	1.892	0.994	1.174	1.386
Abscondence	−0.038	0.367	0.918	−0.103	0.469	0.963	1.978
Duration until revocation of IC	0.053	0.015	<0.001	3.581	1.024	1.054	1.085
Duration of hospitalization	−0.014	0.01	0.142	−1.469	0.968	0.986	1.005
Appeal for prolongation of IC	−0.369	0.584	0.527	−0.633	0.22	0.691	2.17
Appeal for early discharge	0.823	0.344	0.017	2.391	1.16	2.278	4.471
CGI at admission	0.483	0.238	0.043	2.027	1.016	1.621	2.587
ICD-10 secondary F1 diagnosis	0.24	0.319	0.451	0.754	0.681	1.272	2.374

* *Binary logistic regression and ML logistic regression*,

** *ML logistic regression*,

*** *Binary logistic regression*.

The machine learning algorithms (Quadratic SVM, Ensemble RUSBoosted Trees and Logistic regression) predicted the outcome parameters (coercion/no coercion) with a balanced accuracy ranging from 66.5 to 69% (the quadratic SVM algorithm identified 102 out of 170 patients which experienced coercion) in the model with 8 parameters and 71.5–76% in the model with 18 parameters. In contrast the binary logistic regression in SPSS had a balanced accuracy of 68.5% in the 8 item model and 78.5% in the 18 item model. In the 18 item model the logistic regression algorithm identified 121 out of 170 patients which experienced coercion (sensitivity). This resulted in an accuracy of 75%. The binary logistic regression of SPSS identified 124 out of 170 patients which experienced coercion and was more potent in predicting those who did not experience coercion (187 out of 223 patients). This resulted in an accuracy of 78.5%.The Quadratic SVM was able to predict 185 out of 223 patients without coercion and had less potency in predicting the outcome coercion (117 out of 170 patients). For details see Table 5.

**Table 5:** Comparison of the 8 and 18 item models.

	**Quadratic SVM**	**Ensemble RUSBoosted Trees**	**Logistic regression**	**SPSS binary logistic regression**
**8 ITEM MODEL**				
Area under curve	0.74	0.73	0.73	0.75
Balanced accuracy (%) (Specifity + Sensitivity/2)	69	68.5	66,5	68.5
Specificity (%)	78	68	74	76
Sensitivity (%)	60	69	59	61
PPV (%)	68	62	64	67
NPV (%)	72	74	71	72
**18 ITEM MODEL**				
Area under curve	0.78	0.78	0.82	0.86
Balanced accuracy (%) (Specifity + Sensitivity/2)	76	71.5	75	78.5
Specificity (%)	83	74	79	84
Sensitivity (%)	69	69	71	73
PPV (%)	75	67	72	78
NPV (%)	78	76	78	80

*NPV, negative predictive value; PPV, positive predictive value; SVM, support vector machines*.

Due to inconsistent findings in literature we also created two models which did not include the variables gender and substance-use-related disorders as co-diagnosis (which was not significantly associated in our bivariate analyses). The results were comparable but not as robust as the 8 and 18 item model. They are shown in Table 6.

**Table 6:** Comparison of the 6 and 16 item models.

	**Quadratic SVM**	**Ensemble RUSBoosted Trees**	**Logistic regression**	**SPSS binary logistic regression**
**6 ITEM MODEL**				
Area under curve	0.72	0.69	0.73	0.75
Balanced accuracy (%) (Specifity + Sensitivity/2)	69	67	67	69
Specificity (%)	77	63	78	79
Sensitivity (%)	61	71	56	59
PPV (%)	67	59	66	68
NPV (%)	72	74	70	71
**16 ITEM MODEL**				
Area under curve	0.78	0.78	0.82	0.85
Balanced accuracy (%) (Specifity + Sensitivity/2)	75	71	74	77
Specificity (%)	84	73	77	81
Sensitivity (%)	66	69	71	73
PPV (%)	76	66	70	75
NPV (%)	76	75	77	79

*NPV, negative predictive value; PPV, positive predictive value; SVM, support vector machines*.

### Weighting of risk factors to experience coercion

In a next step we analyzed the relevance of each variable in the prediction of the outcome coercion/no coercion. We compared the weights of the included variables between logistic regression in ML and binary logistic regression. We analyzed the relevance of predictor variables in distinguishing the outcome coercion/no coercion. Positive coefficients or weighting factors were assigned to an increase in coercion for the 8 and 18 item models.

In the model with 8 items the CGI at admission had the highest weight. In ML this was followed by the reason for IC, former IC and a police involvement at admission. In binary logistic regression the second weighted predictor was former IC followed by reason for IC and police involvement at admission.

**Figure 1:**
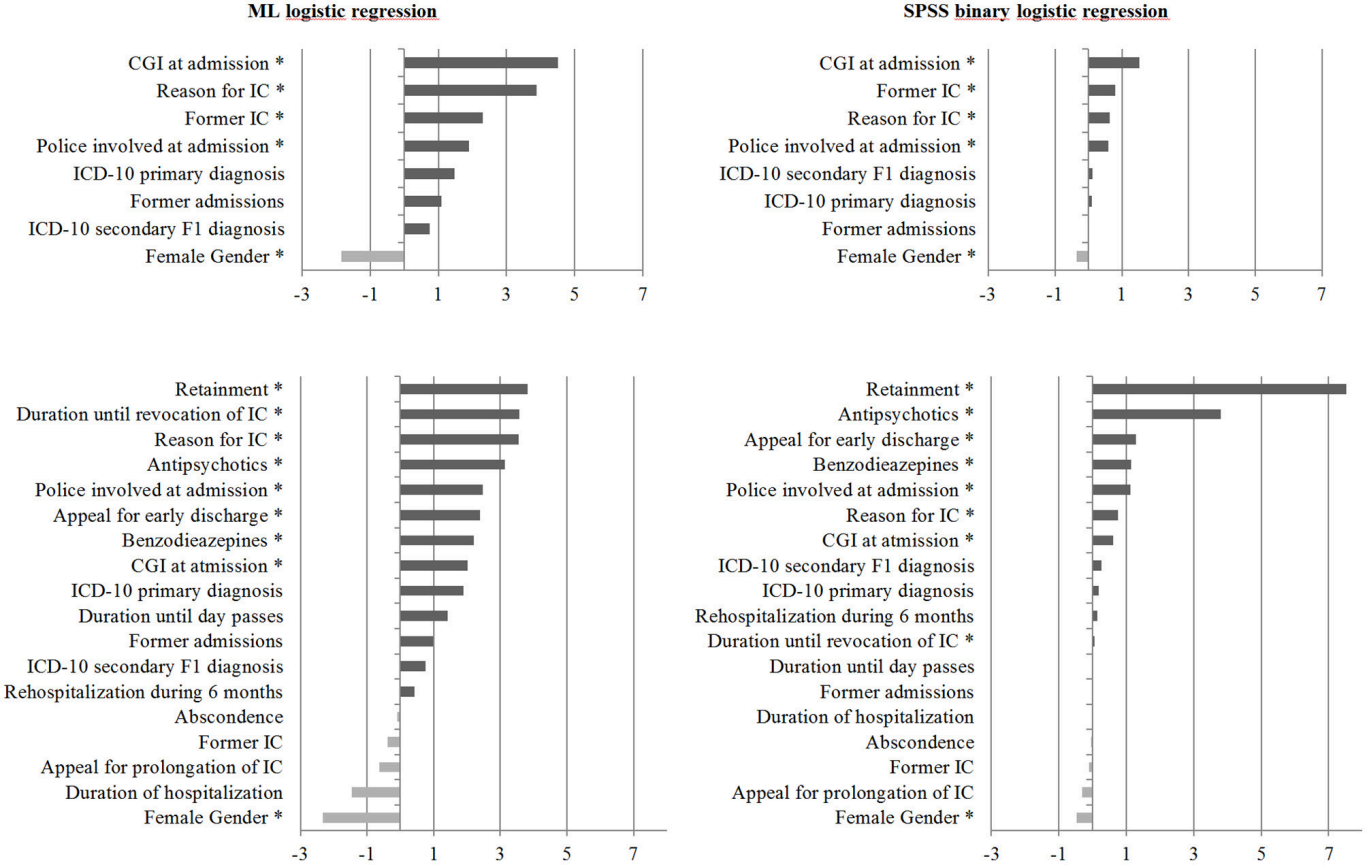
Bar graphs showing weighting factors assigned to each variable based on their relevance in distinguishing the outcome coercion from no coercion. Variables which increase the probability of an individual patient to experience coercion were assigned positive weighting factors whilst those that decrease the probability of a patient experiencing coercion were assigned negative weighting factors. ^*^Significant at the 0.05 level.

In the 18 item model retainment was the highest weighted predictor. In ML this was followed by duration until revocation of IC, reason for IC at admission and prescription of antipsychotic medication. In binary logistic regression antipsychotic medication was weighted after retainment, followed by appeal for early discharge and the prescription of benzodiazepines. In both models female gender was negatively weighted. For details see Figure 1.

## Discussion

This study could show that machine learning algorithms can predict the outcome of coercion/no coercion in a patient group with a good accuracy and have some advantages compared to binary logistic regression which also appeared to have a good accuracy. All algorithms achieved greater than chance (50%) accuracy in distinguishing patients with coercion from those without coercion. We could verify the hypothesis that a model with a higher number of variables (including variables which occur during the course of hospitalization) was more potent in the prediction of the outcome coercion/no coercion. The AUC was acceptable in the model with 8 items with values from 0.73 to 0.75. In the model with 18 items the AUC reached values from 0.78 to 0.86 which implies excellent results in 2 out of 4 algorithms. In the model with 8 items quadratic SVM had the best accuracy whereas binary logistic regression had the best accuracy in the 18 item model. All the included algorithms had a good balance of specificity and sensitivity. Although the binary logistic regression appeared to have a slightly better AUC than the machine learning algorithms the machine learning algorithms appear to have an advantage. By using cross validation the training data are divided into a set of data where the model is trained and another k (in this study *k* = 5) sets of data where the trained model is validated. Thus, the accuracy of the trained model is verified in data sets which are independent of the trained data. This allows better generalizability for the prediction accuracy because it was tested on “new” data. This is different from conventional binary logistic regression where all data are used in one analysis and generalizability is limited.

The fact that the models can predict the occurrence of coercion/no coercion with a good accuracy of 69% in the model with 8 parameters and even more in the model with 18 parameters underlines the relevance of the included variables for clinical use and future research. Although the parameter were not able to explain all variance some of them can be defined as substantial “risk factors” for the experience of coercion during psychiatric hospitalization. In the 8 item model the CGI at admission had the highest weight, followed by reason for IC, former IC, and police involvement at admission. In the 18 item model retainment had the highest weight.

By knowing risk factors and their weights it might be possible to identify groups of patients at risk by using a risk assessment tool. Patients could be divided into different risk groups. Treatment strategies could be adjusted to the different risk groups and help to prevent the occurrence of situations in which the usage of coercion seems necessary. Harm to others as reason for IC, former IC, and police involvement at admission were high weighted in both approaches. Combined with the finding that most coercive measures were applied due to harm to others this implies that aggression is a challenge for staff. This has also been shown in other studies (26, 30, 37–39) and was one reason to develop specialized PICU's where staff is trained in aggression management (40). Retainment, the highest weighted predictor in the 18 item model, implies a high-risk situation and should be considered as a reason for the transfer to such PICUs (15). The CGI, which was highly weighted in the 8 item model is not specific but implies that patients at risk may be more likely to meet the criteria for severe mental illness (SMI). Although being less weighted, the psychiatric diagnosis should also be included in the risk assessment. Patients with a psychotic disorder or a personality disorder appeared to have an increased risk to experience coercion in our analysis and in previous literature (16–26). Also male gender should be considered in the risk assessment. Nevertheless, gender needs to be reflected with caution because other studies found female gender to be significantly associated with coercion (22, 29).

In patients at risk the regular use of the Brøset Violence Checklist could be helpful in identifying situations where the risk for aggressive behavior is increased (41) and was shown to result in a decreased rate of aggressive incidents (42). A cooperation between mental health community and hospital teams (43), personal safety plans or treatment planning (25, 43), single rooms and retreat-rooms on the ward may help avoiding interpersonal stress. As mentioned above it was also shown that the segregation of disruptive patients in a psychiatric intensive care unit (PICU) (44) and the ward atmosphere (35) were effective in the reduction of aggressive behavior. A more recovery orientated view might be helpful to build a relationship between patient and the therapeutic team. Also staff training in communication skills, fast building and maintenance of a stable therapeutic relationship could help to reduce situations in which coercion is used (37).

As mentioned above, previous studies followed different methodological protocols. To provide comparability between different study sites, statistical models should be used which follow a comparable methodological approach. These models should have a good accuracy and be easy to replicate in different countries. This study could show that ML algorithms (logistic regression, SVM, decision trees) can predict the outcome coercion /no coercion in a group of patients with a good accuracy and explain some of the variance. Furthermore machine learning can be used for weighting of the included predictors. Cross validation provides a better generalizability of the results which is attractive for the usage in different study sites. Previous studies could show that beside risk-factors in patients also procedural factors like closed ward doors (45), architecture and atmosphere of a ward (35, 46) or interpersonal factors like escalating behavior of staff (47, 48) may be a risk for violent behavior in the patients and consecutively the usage of coercion. Future studies should therefore aim to analyze the weights of clinical culture, attitude toward coercion in the therapeutic teams and organizational factors to test if these factors account for the unexplained variance in the prediction models used in this study.

## Limitations

Some limitations must be mentioned regarding to this study. Although we runned tests for each predictor alone and different combinations of the predictors some of the predictor variables may influence each other. This may have lead to a bias in the prediction potency of the models.

Artificial balance was created by decreasing the number of participants with the outcome no coercion.

In the group comparison some categories (e.g., diagnostic groups, harm-criteria, CGI-groups) were very small and due to that may have contributed to a significant effect. Previous studies showed comparable findings. On this background we included these small groups in analysis. Further studies should re-evaluate our results with a bigger sample size.

The analysis was based on retrospectively collected data, and it was not possible to assess the subjective perspectives of patients and physicians in a standardized form. Due to the retrospective character of the study the psychopathological symptoms could not be assessed in a standardized way. Because of that, important information about the severity of symptoms during the situation in which coercion was used is lacking. Furthermore it was not possible to assess if alternatives were used before coercion had to be used. We were not able to include data on treatment culture and socio-cultural factors in general into our analysis. This would be an interesting topic for future research.

## Conclusion

This study was able to show that ML is useful in the prediction of coercion and reach comparable results to binary logistic regression although the trained algorithms are used on new sets of validation data (five-fold cross validation) which allows a better generalizability. ML is a promising approach for further research on risk factors and the occurrence of coercion in psychiatry.

Weighting of risk factors may be helpful in the risk-assessment of the individual patients. In patients at risk special therapeutic strategies could be helpful to prevent the occurrence of aggressive behavior and consecutively coercion. Future studies should evaluate the potency of these strategies and the usefulness of risk-assessment tools.

## Ethics statement

The study was reviewed and approved by the Cantonal Ethics committee of Zurich, Switzerland (Ref.-No. EK: 2016-00749, decision on 01.09.2016). Commitment documents as well as the medical records of patients involuntarily hospitalized at the University Hospital of Psychiatry Zurich during a 6-month period from January first to June 30, 2016 were analyzed. All procedures were in accordance with the ethical standards of the institutional and/or national research committee and with the 1964 Helsinki declaration and its later amendments or comparable ethical standards. This is a retrospective study. For this type of study formal consent is not required. This article does not contain any studies with animals performed by any of the authors.

## Author contributions

FH, SO, and MJ: conception and design, data collection, analysis and interpretation of data; FH: drafting the article; FH, AS, AT, PH, ES, SO, and MJ: revising the article critically for important intellectual content; FH, SO, MJ, AS, AT, PH, and ES: final approval of the version to be published.

### Conflict of interest statement

The authors declare that the research was conducted in the absence of any commercial or financial relationships that could be construed as a potential conflict of interest.
